# Multifaceted Analyses of Epidermal Serine Protease Activity in Patients with Atopic Dermatitis

**DOI:** 10.3390/ijms21030913

**Published:** 2020-01-30

**Authors:** Hayato Nomura, Mutsumi Suganuma, Takuya Takeichi, Michihiro Kono, Yuki Isokane, Ko Sunagawa, Mina Kobashi, Satoru Sugihara, Ai Kajita, Tomoko Miyake, Yoji Hirai, Osamu Yamasaki, Masashi Akiyama, Shin Morizane

**Affiliations:** 1 Department of Dermatology, Okayama University Graduate School of Medicine, Dentistry, and Pharmaceutical Science, 2-5-1 Shikata-cho, Kitaku, Okayama 700-8558, Japan; 2 Department of Dermatology, Nagoya University Graduate School of Medicine, 65 Tsurumai-cho, Showa-ku, Nagoya 466-8550, Japan; 3 Department of Dermatology and Plastic Surgery, Akita University Graduate School of Medicine, Hondo 1-1-1, Akita-shi, Akita 010-8543, Japan

**Keywords:** atopic dermatitis, serine proteases, kallikrein-related peptidases, epidermal barrier dysfunction, lympho-epithelial Kazal-type-related inhibitor (LEKTI), *SPINK5*, filaggrin

## Abstract

The serine proteases kallikrein-related peptidase (KLK) 5 and KLK7 cleave cell adhesion molecules in the epidermis. Aberrant epidermal serine protease activity is thought to play an important role in the pathogenesis of atopic dermatitis (AD). We collected the stratum corneum (SC) from healthy individuals (*n* = 46) and AD patients (*n* = 63) by tape stripping and then measuring the trypsin- and chymotrypsin-like serine protease activity. We also analyzed the p.D386N and p.E420K of *SPINK5* variants and loss-of-function mutations of *FLG* in the AD patients. The serine protease activity in the SC was increased not only in AD lesions but also in non-lesions of AD patients. We found, generally, that there was a positive correlation between the serine protease activity in the SC and the total serum immunoglobulin E (IgE) levels, serum thymus and activation-regulated chemokine (TARC) levels, and peripheral blood eosinophil counts. Moreover, the p.D386N or p.E420K in *SPINK5* and *FLG* mutations were not significantly associated with the SC’s serine protease activity. Epidermal serine protease activity was increased even in non-lesions of AD patients. Such activity was found to correlate with a number of biomarkers of AD. Further investigations of serine proteases might provide new treatments and prophylaxis for AD.

## 1. Introduction

Atopic dermatitis (AD) is a chronic, pruritic inflammatory skin disease that affects up to 25% of children and 2–3% of adults [1]. AD has a complex pathogenesis involving genetic, immunologic, and environmental factors which lead to a dysfunctional skin barrier and dysregulation of the immune system [1]. Aberrant epidermal serine protease activity is related to the pathogenesis of inflammatory skin diseases such as Netherton syndrome, AD, psoriasis, and rosacea [2,3,4,5,6,7,8]. Kallikrein-related peptidases (KLKs) are a family of 15 trypsin- or chymotrypsin-like serine proteases encoded by a cluster of protease-encoded genes (*KLK1-15*) in the human genome [9]. KLK5, a trypsin-like serine protease, and KLK7, a chymotrypsin-like serine protease, are major epidermal KLKs. These proteases have roles in the desquamation of epidermis by decomposing cell adhesion molecules such as corneodesmosin, desmoglein 1, and desmocollin 1 [10]. It has been reported that the expression and activity of several KLKs is increased in AD lesions [5,8,11]. Research suggests that KLK5 and KLK7 are particularly involved in the pathogenesis of AD. For instance, KLK5, but not KLK7, directly activates proteinase-activated receptor 2 and induces nuclear factor κB-mediated overexpression of thymic stromal lymphopoietin (TSLP) [12]. Moreover, transgenic-*KLK5* mice display cutaneous and systemic hallmarks of severe inflammation and allergy with pruritus [13]. Transgenic mice expressing human *KLK7* in epidermal keratinocytes have been found to develop pathologic skin changes with increased epidermal thickness, hyperkeratosis, dermal inflammation, and severe pruritus [14]. Our group demonstrated that Th2 cytokines increase the KLK7 expression and function in epidermal keratinocytes, suggesting an association between allergic inflammation and epidermal barrier function [15].

Serine protease activity in the skin is tightly regulated by not only KLKs but also serine protease inhibitors such as lympho-epithelial Kazal-type-related inhibitor (LEKTI), secretory leukocyte protease inhibitor (SLPI), and elafin [16]. LEKTI, encoded by the *serine protease inhibitor Kazal-type 5* (*SPINK5*) gene, is composed of 15 Kazal-type domains, all of which are capable of inhibiting serine protease activity including KLK5 and KLK7 [17,18]. Individuals with mutations of *SPINK5* develop Netherton syndrome, characterized by ichthyosis, hair abnormality, and atopic manifestations [19]. Nonsynonymous variants of *SPINK5* such as the p.D386N (c.G1156A, in exon 13) and p.E420K (c.G1258A, in exon 14) have been reported to be associated with the pathogenesis of AD [20,21,22,23,24,25]. The p.D386N variants disrupt the role of domain (D) 6 of LEKTI which suppresses the induction of TSLP by KLK5 [25]. The p.E420K variant increases the furin cleavage rate at the LEKTI linker region D6–D7 and prevents the formation of the LEKTI fragment D6D9, known to display the strongest inhibitory activity against KLK5-mediated desmoglein 1 degradation [20].

Profilaggrin is dephosphorylated and degraded to produce monomeric filaggrin in the SC and then further proteolyzed to release its component amino acids [26]. Profilaggrin, filaggrin and the amino acids each make different contributions to the epidermal structure and barrier function [26]. In 2006, loss-of-function mutations in the filaggrin gene (*FLG*) were identified as the cause of ichthyosis vulgaris [27]. The same year, it was first reported that loss-of-function genetic variants in *FLG* are predisposing factors in the context of AD [28]. In 2009, it was reported that 27% of Japanese AD patients carried *FLG* mutations [29]. To date, ten *FLG* mutations have been identified in the Japanese population [30]. Although *FLG* mutations are well known to affect epidermal barrier functions, the association between the mutations and the epidermal serine protease activity has not been investigated.

Here, we performed multifaceted analyses of epidermal serine protease activity in patients with AD. We focused particularly on the relationship between the epidermal serine protease activity and biomarkers of AD or AD-related gene variants.

## 2. Results

### 2.1. Serine Protease Activity in the SC of AD Is Increased in Both Non-Lesions and Lesions

**Figure 1:**
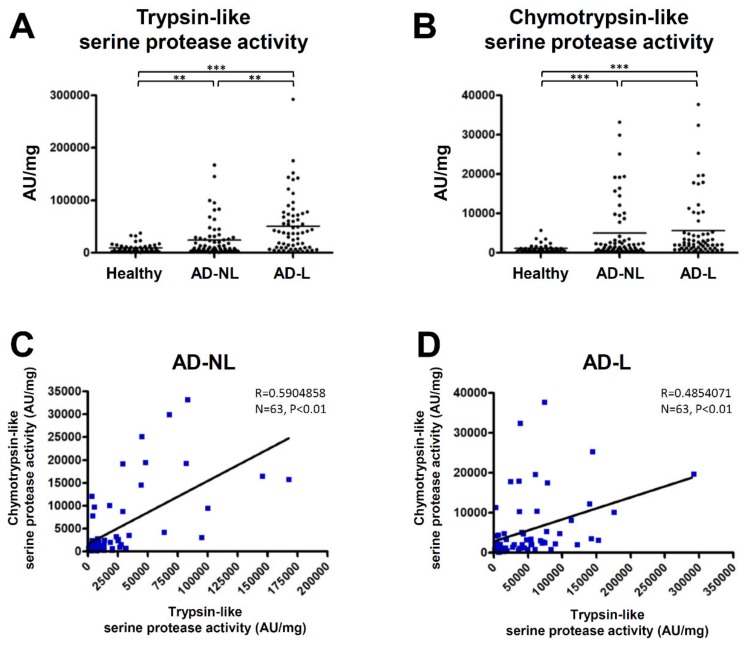
Serine protease activity in the stratum corneum (SC) of atopic dermatitis (AD) patients was increased in both non-lesions and lesions. (**A**,**B**) Trypsin- and chymotrypsin-like serine protease activity in the SC of normal healthy volunteers (*n* = 46) and both non-lesions (AD-NL) and lesions (AD-L) of AD patients (*n* = 63) were measured. ** *p* < 0.01, *** *p* < 0.001. (**C**,**D**) The correlations between trypsin- and chymotrypsin-like serine protease activity in the non-lesions and lesions of AD patients were analyzed by Spearman’s rank correlation coefficient.

We examined trypsin- and chymotrypsin-like serine protease activity in SC samples from normal healthy volunteers and AD patients. The activity of trypsin- and chymotrypsin-like serine proteases in the SC samples from both non-lesions and lesions of the AD patients were significantly higher than those from the healthy individuals (Figure 1A,B). The trypsin-like serine protease activity of the AD lesions was higher than that of the non-lesions (Figure 1A). A significant positive correlation between trypsin- and chymotrypsin-like serine protease activity was observed in both the non-lesions and lesions of the AD patients (Figure 1C,D).

### 2.2. The Association between Serine Protease Activity in the SC of AD and Medical Treatments

**Figure 2:**
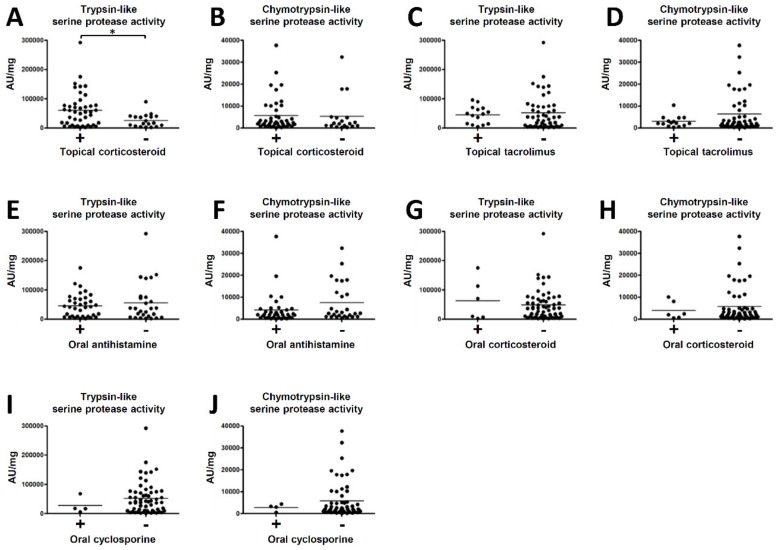
The association between serine protease activity in the SC in lesions of AD patients and medical treatments. Trypsin- or chymotrypsin-like serine protease activity in lesions were compared with and without topical corticosteroid (**A**,**B**), topical tacrolimus (**C**,**D**), oral antihistamine (**E**,**F**), oral corticosteroid (**G**,**H**) and oral cyclosporine therapies (**I**,**J**). * *p* < 0.05.

We next analysed the association between serine protease activity in the SC in lesions of AD patients (*n =* 63) and various medical treatments. The treatments were as follows: topical corticosteroid (*n =* 44), topical tacrolimus (*n =* 15), oral antihistamine (*n =* 36), oral corticosteroid (*n =* 6) and oral cyclosporine (*n =* 4). The trypsin-like serine protease activity in the SC of patients with topical corticosteroid therapy was significantly higher than those without topical corticosteroid therapy (Figure 2A). On the other hand, the chymotrypsin-like serine protease activity in the SC was not significantly different with or without topical corticosteroid therapy (Figure 2B). Neither trypsin- and chymotrypsin-like serine protease activity in the SC was significantly different with or without topical tacrolimus, oral antihistamine, oral corticosteroid or oral cyclosporine therapies (Figure 2C–J).

### 2.3. Serine Protease Activity in the SC of AD Correlate with Biomarkers of AD

**Figure 3:**
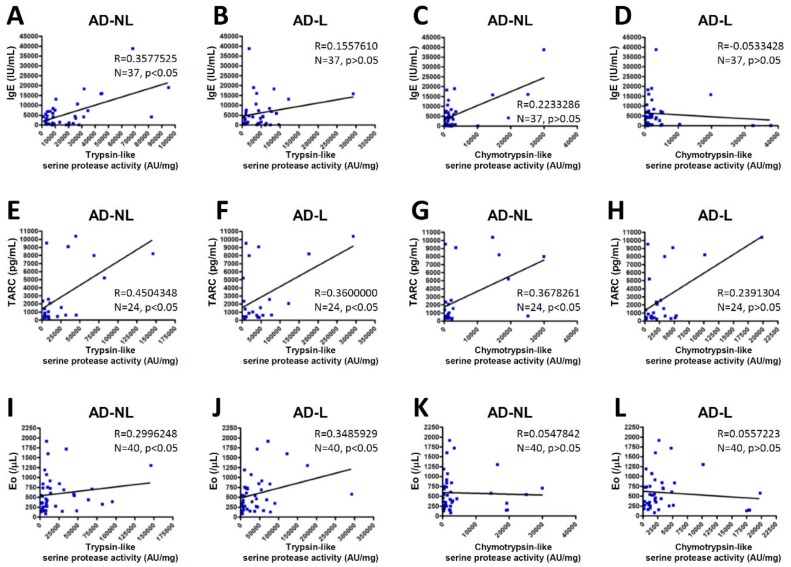
The correlations between serine protease activity in the SC and biomarkers of AD. The correlations between trypsin- or chymotrypsin-like serine protease activity in non-lesions (AD-NL) or lesions (AD-L) and the serum total IgE levels (**A**–**D**), serum TARC levels (**E**–**H**) and the peripheral blood eosinophil counts (Eo) (**I**–**L**) of the AD patients were analyzed by Spearman’s rank correlation coefficient.

We further examined the correlation between serine protease activity in the SC and the biomarkers of AD. We observed that the serum total immunoglobulin E (IgE) levels were highly correlated with the trypsin-like serine protease activity in non-lesions of the AD patients, but not with the chymotrypsin-like serine protease activity in non-lesions or the trypsin- or chymotrypsin-like serine protease activity in AD lesions (Figure 3A–D). The serum levels of thymus and activation-regulated chemokine (TARC) were highly correlated with the trypsin- or chymotrypsin-like serine protease activity in the non-lesions of the AD patients and the trypsin-like serine protease activity in AD lesions, but not with the chymotrypsin-like serine protease activity in AD lesions (Figure 3E–H). Our study found, further, that peripheral blood eosinophil counts were highly correlated with the trypsin-like serine protease activity in the non-lesions and lesions of the AD patients, but not with the chymotrypsin-like serine protease activity in the AD non-lesions or lesions (Figure 3I–L).

### 2.4. p.D386N and p.E420K of SPINK5 and Loss-Of-Function Mutations in FLG Do Not Affect the Serine Protease Activity in the SC

**Figure 4:**
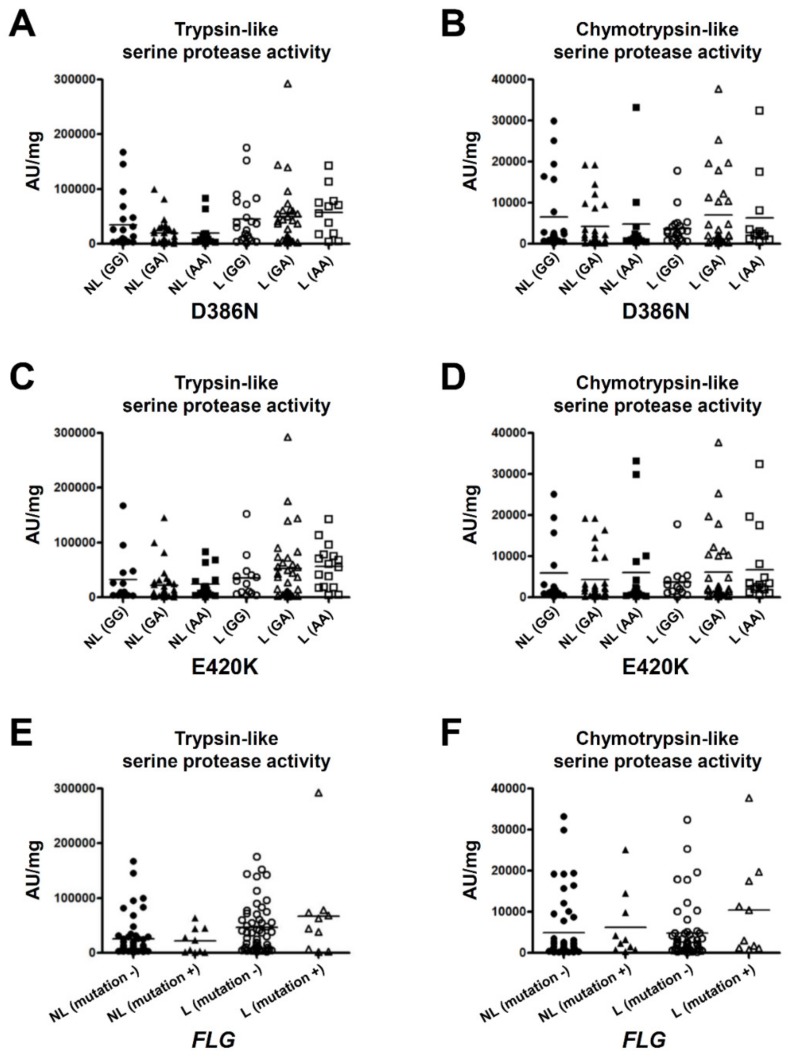
Serine protease activity in the SC of the subgroups of patients with the variants of the *SPINK5* gene and *FLG* gene in AD patients. Trypsin- and chymotrypsin-like serine protease activity in non-lesions and lesions was compared among the subgroups of the p.D386N (**A**–**B**) or p.E420K (**C**–**D**) of *SPINK5* and among the subgroups with or without loss-of-function mutations of *FLG* (**E**–**F**).

To clarify the association between serine protease activity in the SC and the AD-related gene variants, we analyzed *FLG* mutations in the AD patients. Eighteen of the 115 patients (15.7%) had one of the ten *FLG* mutations. We were able to perform protease assays with the SC samples from 61 of the AD patients. Ten of the 61 patients (16.4%) had the *FLG* mutations: p.Q1701X (*n =* 1), p.S2554X (*n =* 1), p.S2889X (*n =* 4), p.S3296X (*n =* 2) and p.K4022X (*n =* 2). We also analyzed p.D386N (c.G1156A) and p.E420K (c.G1258A) of *SPINK5* in the AD patients. In respect of p.D386N, the numbers of the genotypes were as follows: GG (*n =* 21), GA (*n =* 28), and AA (*n =* 12). For p.E420K, the numbers of the genotypes were as follows: GG (*n =* 14), GA (*n =* 31), and AA (*n =* 16). The trypsin- and chymotrypsin-like serine protease activity in the SC was not significantly different among the subgroups of p.D386N (Figure 4A,B) or p.E420K (Figure 4C,D). In addition, *FLG* mutations did not significantly affect the trypsin- or chymotrypsin-like serine protease activity of the SC in the AD patients (Figure 4E,F).

## 3. Discussion

It has been reported that the expression and activity of several KLKs are increased in AD lesions and trigger or enhance epidermal barrier dysfunction [5,8,11]. In this context, we examined the serine protease activity in the SC of both non-lesions and lesions of AD patients. Our findings demonstrated for the first time that serine protease activity is increased not only in lesions but also in non-lesions of AD patients.

Our results are consistent with previous reports that the quantity and activity of serine proteases are increased in AD lesions [5,8]. Our new finding that serine protease activity is also increased in non-lesions suggests two possibilities: (1) Serine protease activity may be influenced by some genetic factors; and (2) Non-lesions of AD patients have subclinical inflammation that may induce the enhancement of serine protease activity.

Considering the fact that Th2 cytokines such as interleukin (IL)-4 and IL-13 increase the expression and function of KLK7 in epidermal keratinocytes [15], the upregulation of chymotrypsin-like serine protease activity may be induced by systemic Th2 inflammation. However, the trypsin-like serine protease activity is not affected by Th2 cytokines [15]. Our present analyses showed that trypsin-like serine protease activity is also increased in both non-lesions and lesions of AD. Further analyses are needed to clarify the mechanism underlying the increase in trypsin-like serine protease activity.

We next investigated the association between serine protease activity in the SC and the content of therapy for AD. The trypsin-like serine protease activity in the SC of patients with topical corticosteroid therapy was significantly higher than those without topical corticosteroid therapy. These results suggest the possibility that patients with clinically severe AD needed to be treated with topical corticosteroid and clinically mild patients were able to be treated with topical tacrolimus or moisturizer only. Thus far, it is unknown why there is a difference between trypsin-like and chymotrypsin-like serine protease activity even though they are positively correlated (see Figure 1). Furthermore, it was unclear whether all the patients strictly took ointments or oral medicines. In addition, we did not prepare a wash-out period before collecting samples. These questions will guide future investigations.

We also investigated the correlation between serine protease activity in the SC and several biomarkers of AD. Not all serine protease activity was significantly correlated with the serum total IgE levels, serum TARC levels, or peripheral blood eosinophil counts, but there were tendencies for these to correlate positively. The absence of statistical significance in several of the comparisons might be due to the insufficiency of the sample number considered for the analysis or the technical instability of our protease assays. Serum total IgE reflects the degree of allergen sensitization [31,32]. Serum TARC is the most sensitive clinical biomarker of AD [33]. Peripheral blood eosinophil numbers also correlate roughly with disease severity [34]. Our results suggest that serine protease activity in the SC correlates with the clinical severity of AD and allergen sensitization due to epidermal barrier dysfunction.

We investigated whether nonsynonymous variants of *SPINK5* and loss-of-function mutations in *FLG* are involved in serine protease activity in the SC. p.D386N and p.E420K polymorphisms of *SPINK5* were each reported to be associated with the pathogenesis of AD [20,21,22,23,24,25]. We found, specifically, that the p.D386N variant disrupts the role of D6 of LEKTI which suppresses the induction of TSLP by KLK5 [25]. Moreover, the p.E420K substitution increases the furin cleavage rate at the LEKTI linker region D6–D7 and prevents the formation of the LEKTI fragment D6D9, known to display the strongest inhibitory activity against KLK5-mediated desmoglein 1 degradation [20]. However, our analyses revealed that p.D386N and p.E420K do not change the trypsin- or chymotrypsin-like serine protease activity of the SC in AD patients. Japanese AD patients might exhibit another genetic particularity related to serine protease activity that neutralizes the effects of p.D386N and p.E420K.

The *FLG* mutations are well known to be strongly associated with the pathogenesis of AD [28,29], but not all AD patients have *FLG* mutations. Therefore, one or more other genetic factors can be expected to be associated with the pathogenesis. We had hypothesized that AD patients without *FLG* mutations have serine proteases-related gene variants such as *SPINK5* and aberrant protease activity, but our analyses revealed that there is no significant association between these mutations and the serine protease activity in the SC. On the contrary, there was a tendency for the AD patients with *FLG* mutations to have higher serine protease activity compared to the patients without *FLG* mutations. Further analyses might clarify the association between the increase in serine protease activity and genetic factors in AD patients.

In conclusion, the results of our present analyses demonstrated for the first time that epidermal serine protease activity is increased even in non-lesions of AD patients and this activity is associated with biomarkers of AD. In addition, p.D386N and p.E420K of *SPINK5* and *FLG* mutations did not affect the serine protease activity of the SC in Japanese individuals with AD. Further investigations of epidermal serine protease activity might enable the design of new therapeutic and prophylactic drugs for the treatment of AD.

## 4. Subjects and Methods 

### 4.1. Human Samples

The SC samples and blood samples were collected with written informed consent from normal healthy volunteers and patients with AD at Okayama University Hospital and its affiliated hospitals. This study was approved by the Ethics Committee of Okayama University (approval nos. 1511-012, 1605-027 and 1809-015).

### 4.2. Collection of the Stratum Corneum

Samples of the SC of healthy individuals were obtained from the forearms of normal healthy volunteers (*n* = 46; ages 35.0 ± 8.8 years). The SC of patients who had been diagnosed with AD based on the diagnostic criteria of Hanifin and Rajka were obtained from the upper extremities (lesions and non-lesions) (*n* = 63; ages 38.2 ± 12.8 years). Cellotape™ (Nichiban, Tokyo) was used to collect the SC samples. The 10-cm-long tape was put on and peeled from the surface of the extremities (approx. 10–20 times) until the adhesion disappeared. The tape was then stored at −20 °C until the following treatment with hexane.

The tape was soaked in 5 mL of hexane and then shaken to detach the SC from the tape’s adhesive film. After the insoluble tape was removed, the solution was centrifuged at 3000 rpm for 15 min. A supernatant was subtracted and 1 mL of hexane was added. A solution was transferred to a 1.5-mL microtube and centrifuged at 15,000 rpm for 15 min. A supernatant was subtracted again and the recovered samples were air-dried. The weight was measured. The dried SC samples were mixed with 200 μL of 10 mmol/L Tris-HCl (pH 7.8) and incubated at 37 °C for 1 h on a shaker and then used in the protease assays.

### 4.3. Protease Assays

For the measurement of protease activity in the SC samples, 100 μL of sample solution was mixed with 100 μL of a substrate that is specific for trypsin-like serine proteases (Boc-Phe-Ser-Arg-MCA; Peptide Institute, Osaka, Japan) or chymotrypsin-like serine proteases (Suc-Leu-Leu-Val-Tyr-MCA; Peptide Institute) in 10 mmol/L Tris-HCl (pH 7.8). The final concentration of each substrate was 0.4 mmol/L. The mixture was incubated at 37 °C for 24 h and the fluorescence (excitation/emission = 380/460 nm) was then measured by a FlexStation 3 Multi-Mode Microplate Reader (Molecular Devices, Sunnyvale, CA) at the Central Research Laboratory, Okayama University Medical School. For the correction of the differences in the SC sample amounts, the value of measured fluorescence (AU) was divided by dried weight of the SC sample (mg).

### 4.4. Blood Examinations and Medical Treatment Histories

The patients’ laboratory data were extracted from their medical records. The serum total IgE level, serum TARC level and peripheral blood eosinophil count that had been obtained at the time point closest to the patient’s tape stripping were selected. We also collected the medical treatment histories of the patients such as their use of topical corticosteroid and topical tacrolimus, oral antihistamine, oral corticosteroid and oral cyclosporine.

### 4.5. Analysis of the SPINK5 Gene and the FLG Gene

Genomic DNA was extracted from whole blood using the Wizard Genomic DNA Purification Kit (Promega, Madison, WI, USA). For exon 13 of *SPINK5*, the 453-bp DNA was amplified with two specific primers (SPINK5-E13-FW: 5′-TTCCTATCTCTTGGCATATGATGT-3′ and SPINK5-E13-RV: 5′-TGTCTCCAATCAGACAGTTTCTC-3′). For exon 14 of *SPINK5*, the 294-bp DNA was amplified with two specific primers (SPINK5-E14-FW: 5′-CAGGGTTAGGCACATCACATTC-3′ and SPINK5-E14-RV: 5′-TAAGGAATGCACGTGTTCCCTG-3′).

The polymerase chain reaction (PCR) products were purified using exonuclease I and shrimp alkaline phosphatase (USB, Cleveland, OH, USA) and sequenced using a BigDye Terminator v3.1 Cycle Sequencing Kit (Applied Biosystems, Foster City, CA, USA) and an ABI3100 DNA sequencer (Applied Biosystems) at the Central Research Laboratory, Okayama University Medical School.

Real-time PCR-based genotyping of *FLG* mutations was performed. In the present *FLG* mutation screening, we investigated the 10 *FLG* mutations as described [30]. Briefly, real-time PCR-based genotyping of the *FLG* mutations was performed with a TaqMan probe genotyping assay. Real-time PCR-based genotyping of the *FLG* mutations was performed with a TaqMan probe genotyping assay according to the manufacturer’s instructions (Roche Diagnostics, Basel, Switzerland). To detect an allele of each mutation, a set of two TaqMan probes labeled with a fluorescent dye (FAM or CAL Fluor Orange 560) and a quencher dye (BHQ-1), in addition to sequence-specific forward and reverse primers, were synthesized by Biosearch Technologies (Novato, CA, USA). The sequence of assay probes/primers was as provided [30]. The real-time PCR was performed with a LightCycler 480 system II 384 plate (Roche Diagnostics) in a final volume of 5 μL. Genotyping was then performed using the endpoint genotyping analysis of the LightCycler 480 software.

### 4.6. Statistical Analyses

All statistical analyses were conducted with GraphPad Prism 4 ver. 4.03 software (GraphPad Software, La Jolla, CA, USA). Student’s *t*-test was used to determine the significance of differences between pairs of groups. The correlation between serine protease activity and the blood examination results was analyzed by Spearman’s rank correlation coefficient. Values of *p* < 0.05 were considered significant.

## Abbreviations

AD	Atopic dermatitis
D	Domain
FLG	Filaggrin
IgE	Immunoglobulin E
IL	Interleukin
KLK	Kallikrein-related peptidase
LEKTI	Lympho-epithelial Kazal-type-related inhibitor
SC	Stratum corneum
SLPI	Secretory leukocyte protease inhibitor
SPINK5	Serine protease inhibitor Kazal type 5
TARC	Thymus and activation-regulated chemokine
Th	T-helper lymphocytes
